# Alterations of lung microbiota in lung transplant recipients with *pneumo*cystis *jirovecii* pneumonia

**DOI:** 10.1186/s12931-024-02755-9

**Published:** 2024-03-14

**Authors:** Qiaoyan Lian, Xiuling Song, Juhua Yang, Lulin Wang, Peihang Xu, Xiaohua Wang, Xin Xu, Bin Yang, Jianxing He, Chunrong Ju

**Affiliations:** 1 State Key Laboratory of Respiratory Disease, National Clinical Research Center for Respiratory Disease, National Center for Respiratory Medicine, Department of Organ transplantation, Guangzhou Institute of Respiratory Health, the First Affiliated Hospital of Guangzhou Medical University, 510120 Guangzhou, Guangdong P.R. China; 2 Vision Medicals Co., Ltd, 510700 Guangzhou, Guangdong P.R. China; 3 Department of Thoracic Surgery, the First Affiliated Hospital of Guangzhou Medical University, 510120 Guangzhou, Guangdong P.R. China

**Keywords:** Lung transplant, *Pneumocystis jirovecii*, Microbiota, Metagenomic next-generation sequencing

## Abstract

**Background:**

Increasing evidence revealed that lung microbiota dysbiosis was associated with pulmonary infection in lung transplant recipients (LTRs). *Pneumocystis jirovecii* (*P. jirovecii*) is an opportunistic fungal pathogen that frequently causes lethal pneumonia in LTRs. However, the lung microbiota in LTRs with *P. jirovecii* pneumonia (PJP) remains unknow.

**Methods:**

In this prospective observational study, we performed metagenomic next-generation sequencing (mNGS) on 72 bronchoalveolar lavage fluid (BALF) samples from 61 LTRs (20 with PJP, 22 with PJC, 19 time-matched stable LTRs, and 11 from LTRs after PJP recovery). We compared the lung microbiota composition of LTRs with and without *P. jirovecii*, and analyzed the related clinical variables.

**Results:**

BALFs collected at the episode of PJP showed a more discrete distribution with a lower species diversity, and microbiota composition differed significantly compared to *P. jirovecii* colonization (PJC) and control group. *Human gammaherpesvirus 4, Phreatobacter oligotrophus*, and *Pseudomonas balearica* were the differential microbiota species between the PJP and the other two groups. The network analysis revealed that most species had a positive correlation, while *P. jirovecii* was correlated negatively with 10 species including *Acinetobacter venetianus*, *Pseudomonas guariconensis*, *Paracandidimonas soli*, *Acinetobacter colistiniresistens*, and *Castellaniella defragrans*, which were enriched in the control group. The microbiota composition and diversity of BALF after PJP recovery were also different from the PJP and control groups, while the main components of the PJP recovery similar to control group. Clinical variables including age, creatinine, total protein, albumin, IgG, neutrophil, lymphocyte, CD3^+^CD45^+^, CD3^+^CD4^+^ and CD3^+^CD8^+^ T cells were deeply implicated in the alterations of lung microbiota in LTRs.

**Conclusions:**

This study suggests that LTRs with PJP had altered lung microbiota compared to PJC, control, and after recovery groups. Furthermore, lung microbiota is related to age, renal function, nutritional and immune status in LTRs.

**Supplementary Information:**

The online version contains supplementary material available at 10.1186/s12931-024-02755-9.

## Introduction

In the recent decades, novel culture-independent techniques for microbial identification have demonstrated that the lungs harbor complex and dynamic communities of microbiota [1]. Lung microbiota communities differ significantly between healthy and diseased states [1]. Analogously, the lung microbiota of transplanted lungs is profoundly altered compared to that of healthy population [2–4]. Dysbiosis of the lung microbiota is associated with pulmonary infection, acute and chronic allograft rejections in lung transplant recipients (LTRs) [5–9]. For instance, the microbiota of LTRs with airway infection is characterized by loss of bacterial diversity [7]; acute rejection is associated with reduced community diversity [8]; and increased lung bacterial burden predicts chronic rejection and death [9]. Synthetically, these data suggest that the lung microbiota composition is a factor influencing the development of complications in LTRs.

Considering the need for daily immunosuppression, LTRs are at a high risk of pulmonary infections, particularly opportunistic pathogens and mixed infections [10]. *Pneumocystis jirovecii* (*P. jirovecii*) is a ubiquitous opportunistic fungus that often colonizes patients with mild immunosuppression or underlying pulmonary diseases, and a shift from *P. jirovecii* colonization (PJC) to pneumonia (PJP) occurs with profound immunodeficiency [11, 12]. *P. jirovecii* infection is prone to be accompanied by multiple infections, particularly with the cytomegalovirus [12–14]. Moreover, it causes lethal pneumonia in LTRs and is associated with a considerable risk of allograft failure [15]. PJP was associated with a 20∼40% mortality rate in individuals with profound immunosuppression, particularly in patients with CD4^+^ T lymphocyte cell counts < 200 cells/mm, and lifelong PJP prophylaxis after lung transplantation is recommended by some experts [16].

A previous study shows that relative to bronchoalveolar lavage fluid (BALF) samples associated with bacterial colonization, those from pneumonia had significantly lower microbial diversity, and was associated with alveolar inflammation and injury [17]. Bacterial burden and community composition of lung microbiota of critically ill patients is altered and predicts clinical outcomes [18]. Although the importance of the lung microbiota of LTRs has been clarified, few studies have described the lung microbiota in LTRs with fungal infections. A recent study suggested bacterial diversity at the onset of invasive pulmonary aspergillosis predicted the survival of infected patients, and highlight lung microbiota as a potential diagnostic and therapeutic target in the context of respiratory fungal diseases [19]. The adherence of *P. jirovecii* to alveolar epithelium may not only result in diffuse alveolar damage, but also induces host’s inflammatory response that causes significant lung injury. Besides, interactions between individual bacteria and fungi have been reported [20]. Heretofore, no study has determined whether the lung microbiota is altered in patients between PJP and PJC, and whether it is associated with clinical variables.

The aim of the present study was to analyze microbial communities in BALF collected from LTRs with and without *P. jirovecii* using metagenomic next-generation sequencing (mNGS). We characterized and compared the lung microbiota composition of LTRs with and without *P. jirovecii*, as well as the related clinical variables.

## Materials and methods

### Study design and participants

In this prospective observational study, we collected 72 BALFs from 61 LTRs (20 with PJP, 22 with PJC, 19 time-matched stable LTRs, and 11 from LTRs after PJP recovery) who had visited the First Affiliated Hospital of Guangzhou Medical University between August 2020 and April 2023. Clinical information collected from LTRs included demographics, transplant information, maintenance of immunosuppressive agents after lung transplantation, prophylaxis for PJP, laboratory examinations, imaging manifestations, clinical diagnosis, therapeutic regimens, and patient outcomes.

PJP was diagnosed according to the 2020 criteria of the European Organization for Research and Treatment of Cancer and the Mycoses Study Group Education and Research Consortium [21]. Subsequently, standard doses of trimethoprim-sulfamethoxazole (TMP-SMX; 15 mg/kg/day of TMP, PO, divided every 6 h) were initially prescribed and then gradually reduced according to the patient’s condition. Although the lung lesions were completely absorbed, a single tablet (80 mg of TMP plus 400 mg of SMX) was administered once daily for lifelong prophylaxis to prevent PJP recurrence. The PJC group included LTRs with positive results for *P. jirovecii* detection in BALF but no evidence of pneumonia. For LTRs with PJC, we prescribed a single tablet (80 mg of TMP plus 400 mg of SMX) once daily for lifelong PJP prophylaxis. The control group included LTRs with no evidence neither of *P. jirovecii* in BALF samples nor respiratory abnormalities. The control group comprised randomly selected LTRs scheduled for routine surveillance bronchoscopy at around the same time as those with PJP or PJC to obtain an approximately 1:1 case–control ratio.

The study procedures were conducted in accordance with the tenets of the Declaration of Helsinki. The study protocol was approved by the Ethics Review Committee of the First Affiliated Hospital of Guangzhou Medical University (No. 128, 2020). Patient approval and informed consent were obtained from each patient. Written informed consent was obtained from the donors who were alive or from the family members of donors who had died. All donors donated the organs after cardiac or brain death.

### Immunosuppressive regimen

The immune suppressants of LTRs in this study including tacrolimus, mycophenolate mofetil, and prednisone. The doses of tacrolimus were adjusted according to whole-blood concentrations to achieve target C_0_ levels of 13∼19 ng/ml during the perioperative period, 10∼15 ng/ml within 1 year post lung transplant, and 5∼10 ng/ml beyond the first year. Mycophenolate mofetil (500 mg, twice daily) was routinely administered postoperatively, and subsequent doses were adjusted according to the white blood cell count and immune state. Prednisone dose was gradually tapered down daily to 0.25 mg/kg over 1 week after lung transplantation and then maintained for 1 year, and 0.1∼0.15 mg/kg/day beyond the first year.

For LTRs diagnosed with PJP, tacrolimus concentration was reduced to half or less for 7∼14 days and mycophenolate mofetil was withdrawn for 14∼21 days or more according to the immune state. In addition, prednisone (40∼60 mg, twice-daily) was administered for 5∼7 days, with a gradual reduction in dose over a further 7∼14 days in order to prevent immune reconstitution pneumonitis [22]. For the PJC group, we reduced the dose of mycophenolate mofetil, maintained the tacrolimus trough level within target range, and holded the dose of prednisone as usual.

### Sample collection

Most BALF samples (52/72, 72.22%) were collected from the LTRs after the first postoperative year. Experienced bronchoscopists performed the BAL procedure following a standardized protocol. Normal saline (60∼80 mL) was injected at a target recovery rate of 40%∼60%. For LTRs with PJP, BALF samples were collected before the anti-*P. jirovecii* treatment from the transplanted lung lobes with the most prominent lesions on chest computed tomography. For LTRs without PJP, BALF samples were collected from the transplanted lower lung lobes after routine surveillance bronchoscopy. In this study cohort, 65.00% (13/20) LTRs in PJP group were receive antibiotics and 45.00% (9/20) were receive anti-virus medication before sampling, while no antibiotics and anti-virus medications were prescribed in PJC and control groups within the previous 4 weeks. After anti-*P. jirovecii* treatment completion and complete absorption of lung lesions, BALF samples were collected from the previously infected transplanted lung lobes. The BALF samples were immediately stored in sterilized containers, transferred to a laboratory for sequencing within 2 h, and stored at 4 °C.

### Nucleic acid extraction, library preparation, and sequencing

DNA was extracted from all samples using the QIAamp® UCP Pathogen DNA Kit (Qiagen), following the manufacturer’s instructions. Human DNA was removed using Benzonase (Qiagen) and Tween20 (Sigma) [23]. Libraries were constructed for DNA using the Nextera XT DNA Library Prep Kit (Illumina, San Diego, CA) [24]. The library quality was assessed using the Qubit dsDNA HS Assay kit followed by a high-sensitivity DNA kit (Agilent) on the Agilent 2100 Bioanalyzer. Library pools were then loaded onto the Illumina NextSeq 550Dx sequencer for 75 cycles of single-end sequencing to generate approximately 20 million reads for each library. For negative controls, we prepared swabs from 10 healthy donors and added 10^5^ HeLa cells/mL using the same protocol. Sterile deionized water was extracted with specimens to serve as non-template controls [24, 25].

### Bioinformatics analyses

Trimmomatic [26] was used to remove low-quality reads, adapter contamination, duplicate reads, and reads shorter than 50 bp. Low-complexity reads were removed using Kcomplexity with default parameters [27]. Human sequence data were identified and excluded by mapping to a human reference genome (hg38) using the Burrows–Wheeler Aligner software [28]. After alignment, multiple indicators were comprehensively evaluated to obtain a list of suspected microorganisms. Accordingly, microbiota composition profiles were inferred from quality-filtered forward reads with Kraken V.2.1.2 and Bracken V.2.6.2 using the k2_pluspf_20210517 database. For microorganisms detected in negative controls, the mean and standard deviation values of the reads per million (RPM) were calculated, and the RPM (mean + two standard deviations) was set as a positive cutoff for filtering contamination or kitome.

### Statistical analysis

R-Base V.4.1.0 was used to enter the site by species counts and relative abundance tables for statistical analysis. Using the Vegan package in R (version 2.5.7), the alpha diversity of the microbiota profile for each individual was evaluated by group at various data points. The samples’ genus- or species-level compositional profiles were shown through the use of principal component analysis (PCA) ordinations. Using the Vegan package in R (version 2.5.7), permutational multivariate analysis of variance and PCA statistics were used to evaluate the various groups. The function dudi.pco in the ade4 package in R (version 1.7.18) stated the PCA results. Using linear discriminant analysis (LDA) effect size, associations between particular microbial species or genera and patient parameters were found [29]. We employed a multi-scale network analysis approach (Igraph) and Cytoscape to construct a network diagram in order to detect possible microbe-microbe relationships. CCA analysis is a multivariate analysis technique that can link alterations in clinically significant markers to the makeup of microbes. The maximum Pearson correlation coefficient of the difference between clinical indicators and the sample community’s distribution was determined using the “cca” function of the Vegan software package. The maximum correlation coefficient was then utilized to choose the subset of clinical indicators. Next, CCA was applied to the clinical indicators and the sample species distribution table, respectively. The significant *P*-value, or probability greater than or equal to the R2 value, was computed. To acquire the data distribution of the degree of correlation between each clinical indicator and the ranking axis, the data were jumbled with the “envfit“ [18, 30] function (permutation test = 999). The aforementioned processes were then repeated to obtain the data distribution of the degree of correlation between each clinical indicator and the ranking axis.

## Results

### Patient cohort and samples

A total of 72 BALF samples collected from 61 LTRs were sequenced and analysed. Table 1 shows the demographics and underlying clinical conditions of the three groups. Among them, 34 (55.74%) LTRs underwent unilateral lung transplantation, while 27 (44.26%) underwent bilateral lung transplantation. The most prevalent primary indication for lung transplantation was interstitial lung disease (29/61, 47.54%), followed by chronic obstructive pulmonary disease (14/61, 22.95%). All LTRs received a standard triple protocol for immune suppression, including tacrolimus, mycophenolate mofetil, and steroids, without TMP-SMX for PJP prophylaxis or azithromycin for chronic rejection after lung transplantation. Details on antibiotic and anti-virus medications of LTRs in PJP group before sampling are presented in Table S1. In this population, PJP occurred in the transplanted lungs.

**Table 1:** Demographics and clinical characteristics of the study cohort

Patient characteristics	PJP (*n* = 20)	PJC (*n* = 22)	Control (*n* = 19)	*p*-value
Age (years, mean ± SD)	52.90 ± 17.33	55.32 ± 14.81	51.95 ± 11.11	0.749
BMI (kg/m^2^, mean ± SD)	21.09 ± 3.74	20.69 ± 3.09	20.64 ± 3.23	0.644
Sex, n(%)				0.865
Male	15(75.00)	15(68.18)	13(68.42)	
Female	5(25.00)	7(31.82)	6(31.58)	
Primary disease, n(%)				0.827
COPD	4(20.00)	7(31.82)	3(15.79)	
ILD	9(45.00)	10(45.45)	10(52.63)	
Pneumoconiosis	3(15.00)	1(4.55)	3(15.79)	
Others*	4(20.00)	4(18.18)	3(15.79)	
Type of lung transplantation, n(%)				0.433
Single	13(65.00)	10(45.45)	11(57.89)	
Bilateral	7(35.00)	12(54.55)	8(42.11)	
Postoperative duration, Median (P_25_, P_75_), days	463(316, 851)	570(253, 731)	566(317, 712)	0.998

BMI, body mass index; COPD, chronic obstructive pulmonary disease; ILD, interstitial lung disease; SD, standard deviation

*Other primary disease including bronchiectasis, paraquat lung, pulmonary arterial hypertension, pulmonary alveolar proteinosis, Langerhans cell histiocytosis, lymphangioleiomyomatosis, and bronchiolitis obliterans syndrome

### Pulmonary microbial composition and diversity in the three groups

**Fig. 1:**
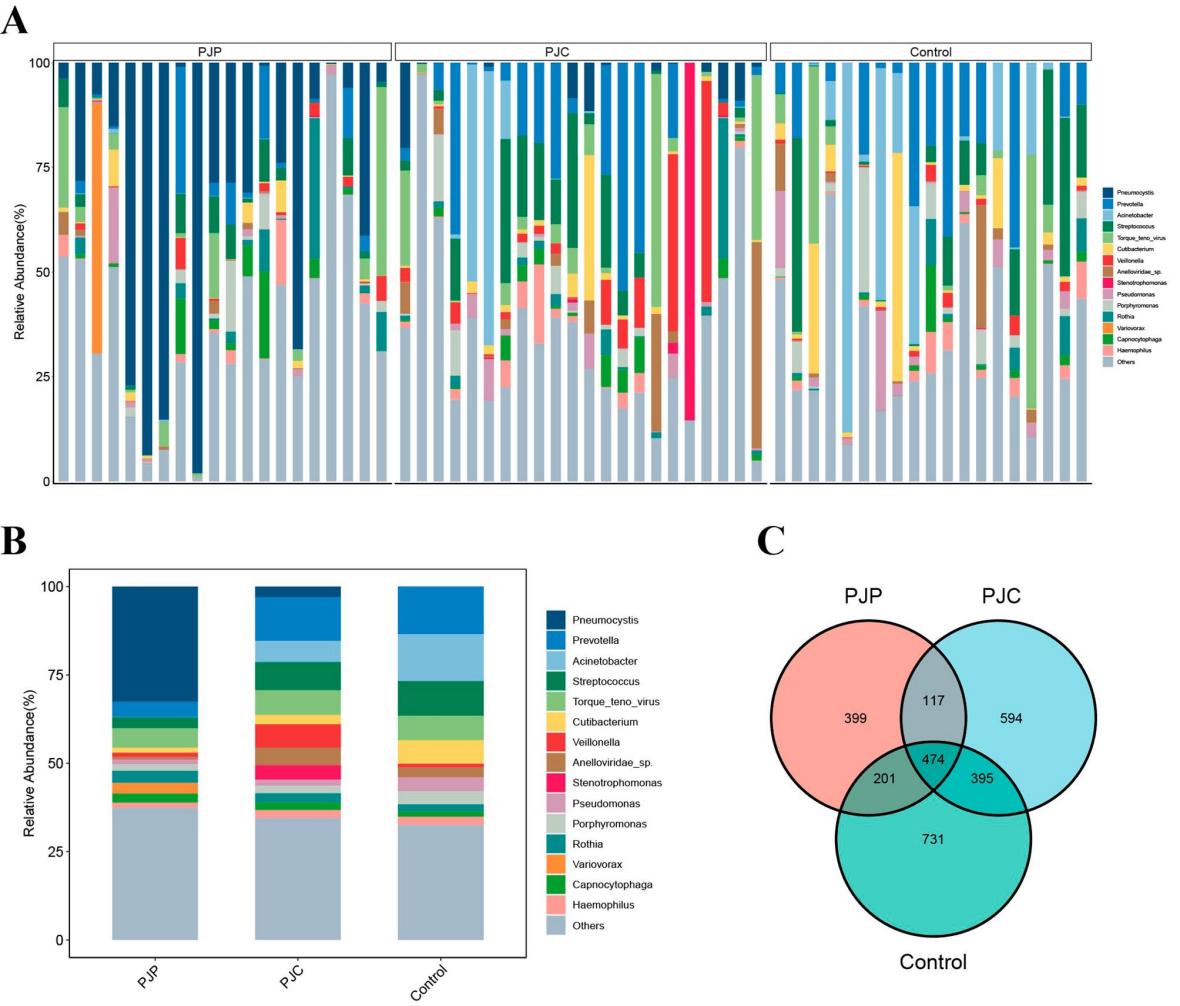
The overall structure of lung microbiota in LTRs. (**A**) Relative abundance of the 15 most abundant microbiota at the genus level in BALF of LTRs. (**B**) Relative abundance of microbiota at the genus level in PJP, PJC and control groups. (**C**) Venn diagram of shared and independent species in the three groups

Using mNGS, we first analyzed the microbiota composition of 61 BALF samples from 61 LTRs, including 20 with PJP, 22 with PJC, and 19 time-matched controls. Figure 1A illustrates the distribution of the top 15 microbial genera with relative abundances in each sample. In the overall cohort, *Prevotella*, *Acinetobacter*, *Streptococcus*, *Torque teno virus*, and *Cutibacterium* were the most abundant genus in addition to *Pneumocystis* (Fig. 1B). The three groups shared 474 microbial species; however, 399, 594, and 731 species were specifically detected in the PJP, PJC, and control groups, respectively. Compare to control group, there were fewer unique species in PJP and PJC groups (Fig. 1C).

**Fig. 2:**
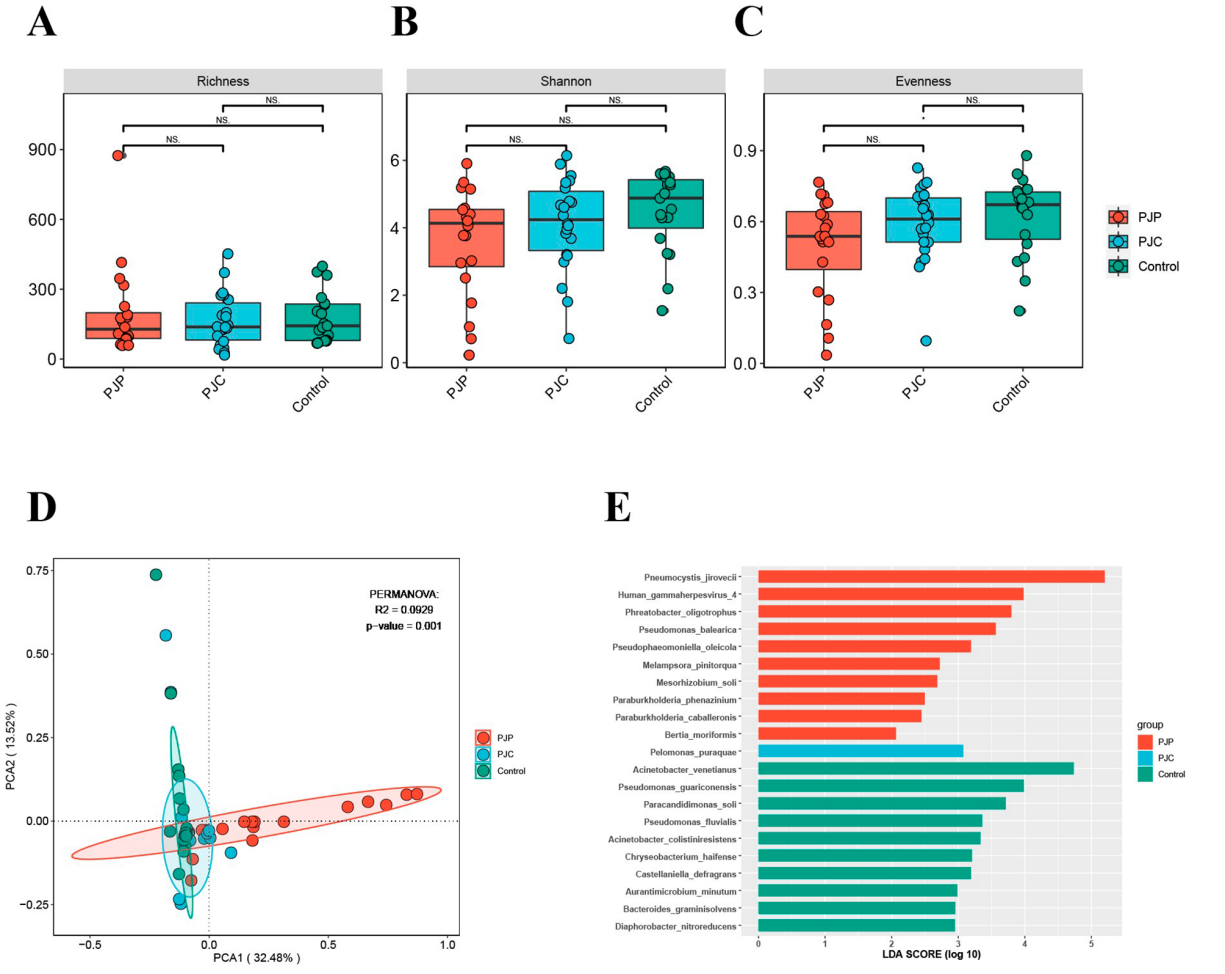
Microbiota community diversity and composition in PJP, PJC and control groups. Alpha diversity of the lung microbiota in the three groups, measured by the Richness (**A**), Shannon diversity index (**B**), and Evenness (**C**). (**D**) PCA of microbial communities showing that the community composition of lung microbiota was distinct in BALF from LTRs in the three groups. (**E**) Histogram of the LDA value distribution of significantly different species among the three groups (LDA score > 2.0 with *p* < 0.05)

Next, we compared the diversity of the pulmonary microbiota in LTRs with and without *P. jirovecii*. Alpha diversity metrics within each sample was assessed by Richness, Shannon diversity index, and Evenness. There was no statistically significant difference of alpha diversity among the three group (*p* > 0.05, Fig. 2A-C). However, the microbiota of BALF samples collected from the PJP group showed a more discrete distribution and had a lower Shannon diversity index and Evenness, indicating a lower species diversity. The microbiota composition differed significantly when BALFs collected at the episode of PJP were compared to non-infected samples using PCA based on the Bray–Curtis distance (Fig. 2D, *p* < 0.05).

### Microbiota taxon and different microbial interaction of LTRs with and without *P. jirovecii* in BALF

To identify the specific taxa responsible for differences in community composition among the three groups, we applied a random forest model to screen for prominent taxa. As expected, *P. jirovecii* contributed the most to microbial differences between the PJP and other groups. In addition to *P. jirovecii, Human gammaherpesvirus 4, Phreatobacter oligotrophus*, and *Pseudomonas balearica* were the differential microbiota species between the PJP and the other two groups. While *Pelomonas puraquae* was the only differential microbiota species in the PJC group. *Acinetobacter venetianus, Pseudomonas guariconensis, Paracandidimonas soli, Pseudomonas fluvialis*, and *Acinetobacte colistiniresistens* contributed to the microbial differences between control and the other two groups (LDA score > 2.0 with *p* < 0.05, Fig. 2E, Figure S1).

**Fig. 3:**
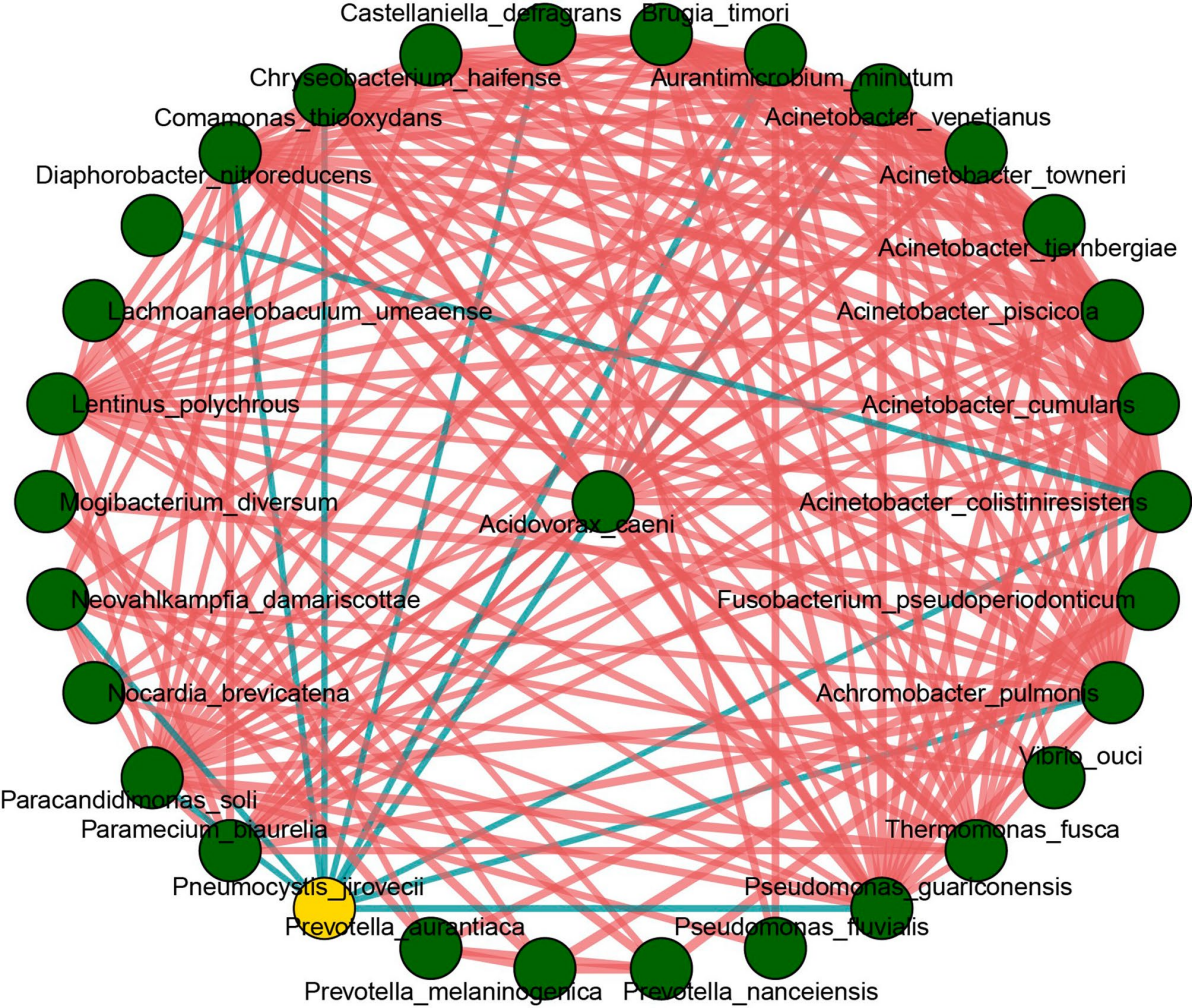
Network correlation of pulmonary microbiota of the study samples. Yellow point represents the *P. jiroveci* and the green points represent other genera. The red line represents positive correlation, the blue line represents negative correlation

The transplanted lung might have a high potential for polymicrobial interactions, so Spearman correlations between *P. jirovecii* and other species were further observed in a correlation network (Fig. 3). The correlation was calculated for all species in the sample, and the flora with *p*value or padj < 0.05 and the absolute value of correlation > 0.4 were selected for display. 31 species were correlated and most of them had a positive correlation, while *P. jirovecii* was correlated negatively with 10 species including *Acinetobacter venetianus*, *Pseudomonas guariconensis*, *Paracandidimonas soli*, *Acinetobacter colistiniresistens*, and *Castellaniella defragrans*, which were enriched in the control group (Fig. 2E), indicating that the presence of *P. jiroveci* might disturb the microbial ecosystems of the lungs.

### Lung microbiota in LTRs with PJP and after PJP recovery

**Fig. 4:**
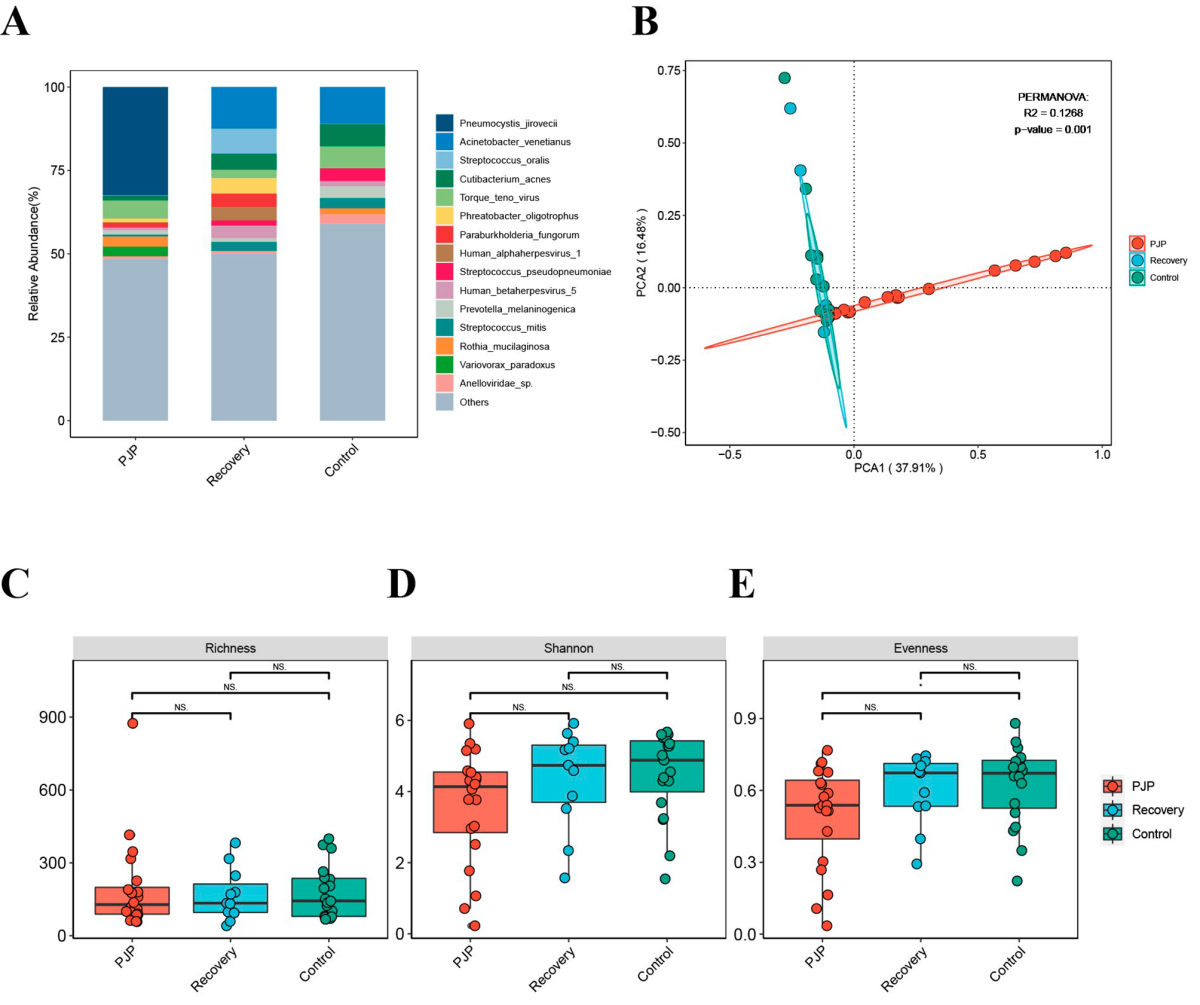
Microbiota composition and community diversity of the study samples. (**A**) Relative abundances of microbiota at the species level in LTRs of PJP, after PJP recovery, and control groups. (**B**) PCA of microbial communities revealed that the community composition of lung microbiota was distinct in BALF from LTRs among the three groups. (**C, D** and **E**) Alpha diversity of the lung microbiota in the three groups, measured by Richness (**C**), Shannon (**D**), and Evenness (**E**) indices

Eleven BALF samples were collected from LTRs diagnosed with PJP and fully cured after anti-PJP treatment. Figure S2 respectively shows the distribution and composition of the 15 most abundant microbiota genera in LTRs with PJP and after PJP recovery. *Acinetobacter venetianus*, *Streptococcus oralis*, *Cutibacterium acnes*, and *Phreatobacter oligotrophus* were the most abundant species after PJP recovery, which were also different to the control group (Fig. 4A). Alterations in the lung microbiota were further confirmed using the LDA effect size method. We detected a total of 28 key phylotypes whose relative abundances significantly different in the lung microbiota of PJP, after PJP recovery and control groups. *P. jirovecii* was barely detected in the PJP recovery group, which was characterized by a higher abundance of *Acinetobacter venetianus, Phreatobacter oligotrophus, Paraburkholderia fungorum, Human gammaherpesvirus 4, and Paraburkholderia ferrariae* (LDA score > 2.0 with *p* < 0.05, Figure S3). *Human gammaherpesvirus 4* was enriched in PJP, PJC and after PJP recovery groups (Figure S1, Figure S3), this phenomenon suggested that immunity was lower than that of the control group.

The alpha diversity index showed no significant differences among PJP, recovery and control groups. However, the Shannon and Evenness indices were lower in PJP group (Fig. 4C–E). The microbiota composition differed significantly when BALF samples collected at the episode of PJP were compared to PJP recovery and control groups using PCA based on the Bray–Curtis distance. The main components of the PJP recovery group differed from those of the PJP group, but similar to the controls (Fig. 4B).

### Lung microbiota was correlated with clinical parameters

**Fig. 5:**
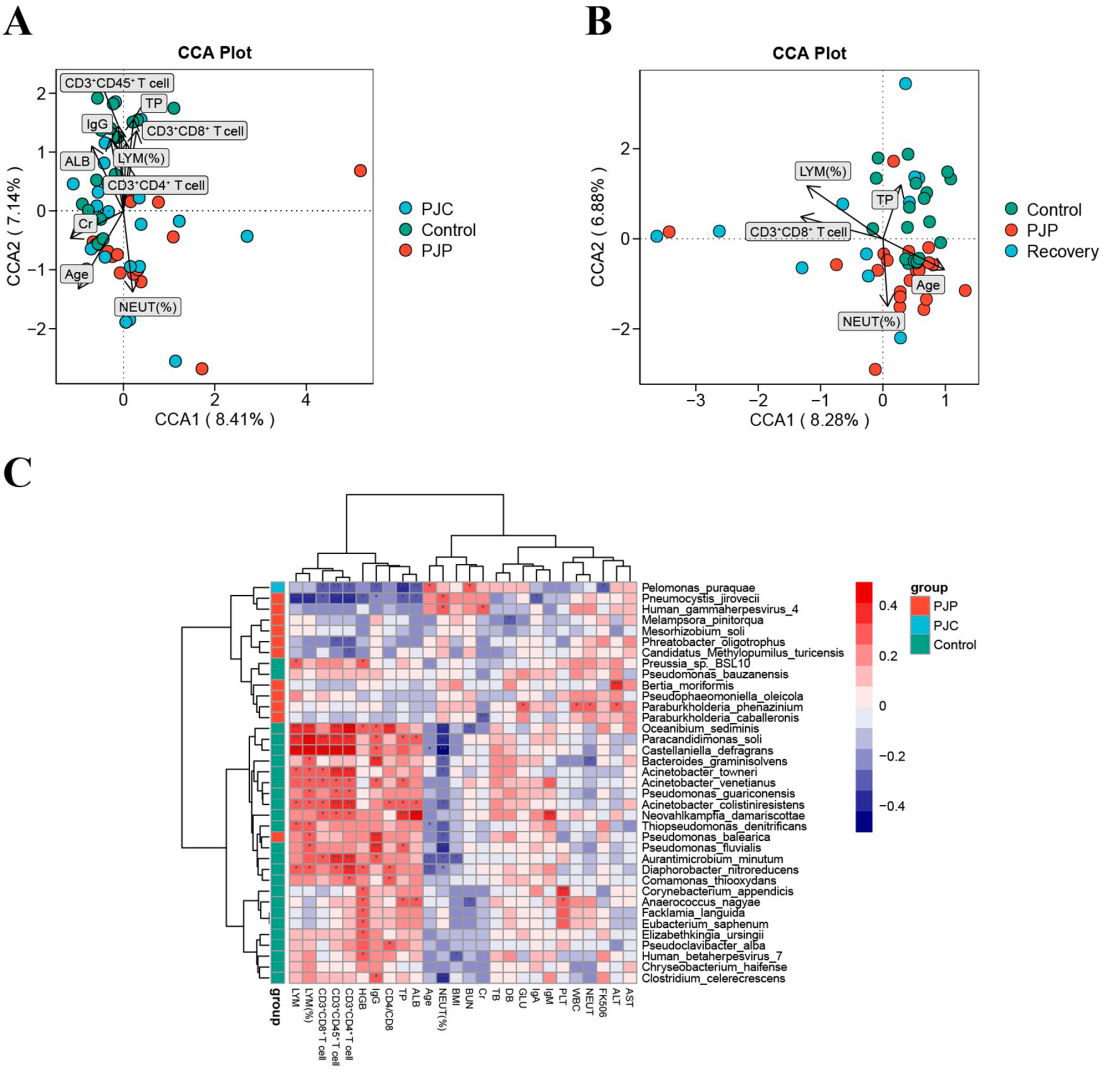
CCA analysis of the relationship between microbial community and clinical parameters. Correlation analysis between the microbiota and clinical parameters among PJP, PJC, and control groups (**A**); PJP, control, and after PJP recovery groups (**B**). (**C**) Heatmap of correlations between lung microbiota and clinical parameters. Red represents a positive correlation, and purple indicates a negative correlation. Black stars within heatmap boxes indicate significant results (*p < 0.05). ALB, albumin; ALT, alanine aminotransferase; AST, aspartate transferase; BMI, body mass index; BUN, blood urea nitrogen; Cr, creatinine; DB, direct bilirubin; FK506, tacrolimus; GLU, glucose; HGB, hemoglobin; LYM, lymphocyte; NEUT, neutrophil; PLT, platelet; TB, total bilirubin; TP, total protein; WBC, white blood cell

We also investigated the possibility of a relationship between lung microbiota and clinical variables of LTRs with PJP. Canonical correlation analysis (CCA) was used to evaluate the influence of clinical parameters on our sample distribution. It is worth noting that creatinine (Cr), age, neutrophil percentage (NEUT%) were positively correlated to PJP BALF microbiota, while lymphocyte percentage (LYM%), albumin (ALB), total protein (TP), IgG, CD3^+^CD45^+^, CD3^+^CD4^+^, and CD3^+^CD8^+^ T cells were negatively correlated (Fig. 5A). Figure 5B showed that CD3^+^CD8^+^ T cell, LYM% and TP were negatively correlated to PJP group, while positively correlated in the control group. However, the recovery group was discrete distributed in the four quadrants, which reflected week correlation between lung microbiota and clinical variables after PJP recovery.

We further performed a Spearman correlation analysis of altered microbiota and 26 clinical parameters. The relationship between lung microbiota and clinical indices was showed in heatmap (Fig. 5C). Over 30 genera had correlation with at least one clinical variable. *Paracandidimonas soli*, *Castellaniella defragrans*, *Bacteroides graminisolvens*, *Acinetobacter venetianus*, and *Acinetobacter colistiniresistens* which enriched in the control group (Fig. 2E) were positively correlated with clinical parameters, such as LYM, IgG, ALB, TP, CD3^+^CD45^+^, CD3^+^CD4^+^ and CD3^+^CD8^+^ T cells. Other species that enriched in PJC and PJP groups (Fig. 2E), such as *Pelomonas puraquae*, *P. jirovecii*, and *Human gammaherpesvirus 4* had negative relationship with the above indicators and positive related to the age and NEUT%. These observations suggested the dynamics of lung microbiota is associated with the physical conditions and immune status of LTRs.

## Discussion

This prospective pilot study demonstrated a distinct characterization of lung microbiota in LTRs with and without *P. jirovecii* infection. The PJP, PJC, and control groups differed significantly in microbiota composition, and PJP were characterized by lower species diversity. The microbiota composition and diversity of BALFs after PJP recovery were also different from the PJP and control groups, while the main components of the PJP recovery was similar to controls. For the microbial interactions, we found that *P. jirovecii* had a negative correlation with other species, which were enriched in the control group, suggested that the presence of *P. jirovecii* is associated with a disruption of the homeostasis of the lung microbiota, causing bacterialimbalance. Furthermore, clinical variables including age, Cr, TP, ALB, IgG, neutrophil, lymphocyte, CD3^+^CD45^+^, CD3^+^CD4^+^ and CD3^+^CD8^+^ T cells were deeply implicated in the alterations of lung microbiota in LTRs, which indicated lung microbiota is related to age, renal function, nutritional state, and host immunity.

The lung microbiota of a healthy population is characterized by low bacterial load and high species diversity, with the most common genera being *Prevotella*, *Streptococcus*, *Veillonella*, *Neisseria*, *Haemophilus*, and *Fusobacterium* [7, 31]. The lung microbiota in LTRs differ in structure and composition from those in healthy subjects, representing a lower microbial diversity and richness but a higher bacterial burden [4, 32]. LTRs have lower abundances of *Prevotella*, *Streptococcus*, *Veillonella*, and *Gemella* than non-transplant populations that dynamically changes with disease states [4, 32, 33]. In our study, *Prevotella*, *Acinetobacter*, and *Streptococcus* were the most abundant genera in the PJC and control groups and differed from that in the PJP group, indicated a state of dysbiosis in the lung microbiota of LTRs with PJP. The microbiota composition differed significantly in groups with and without *P. jirovecii* infection, consistent with previous studies showing that the lung microbiota composition differed significantly between infection, colonization, and non-infection cases [7, 17, 34].

In addition, the PJP group had a lower alpha diversity, although no difference was observed compared to the PJC and control groups. The alpha diversity of the lung microbiota in the PJP group showed a more discrete distribution, suggesting weak consistency among different individuals in the PJP group. These findings were in accordance with those of Lowe et al.’s [35] and our experience that the clinical condition of patients with PJP was complex and always coinfected with other pathogens. The prevalence of PJC is particularly high in patients with chronic pulmonary diseases and also a risk factor for the development of pulmonary diseases [14, 36, 37]. The lung microbiota composition from PJC was similar to the controls in our study, while a shift from PJC to PJP would occur with profound immunodeficiency [11, 12]. Whether or not the lung microbiota in LTRs with PJP results from the interactions between microbes with the host immune system requires further investigation. This will be of significance for understanding PJP pathophysiology more comprehensively, and discerning between *P. jirovecii* infection and carriage.

Given the need for daily immunosuppression, LTRs could be particularly susceptible to changes in the lung microbiota. As an opportunistic pathogen responsible for severe pneumonia in patients with immunocompromise, over 50% of patients with PJP concomitantly have viral or bacterial pneumonia [38]. As is well known, PJP is prone to be accompanied by cytomegalovirus. However, we found that *Human gammaherpesvirus 4* was enriched in BALF of LTRs with PJP and PJC, which revealed immunodeficiency in these patients. From the microbial interactions, we found that most species correlated positively with each other, but *P. jirovecii* correlated negatively with 10 species, which were enriched in the control group, indicated that the presence of *P. jirovecii* destroy the balance of the lung microbiota, causing bacterial imbalance. Our results are consistent with the relationship among members of the lung microbiota, such as mutualism (positive interactions) and antagonism (negative interactions) [6, 39, 40]. It seems that it might be the host microenvironment instead of *P. jirovecii* directly promoted the growth of the bacterial community.

TMP-SMX is recommended for PJP prophylaxis in LTRs for at least 6∼12 months post-lung transplantation, and for LTRs with a history of PJP, lifelong prophylaxis may be indicated [41]. At our lung transplant center, TMP-SMX is not generally prescribed for PJP prophylaxis, but LTRs with a previous episode of PJP are on lifelong prophylaxis to prevent recurrence. In this study, the main components after PJP recovery was significantly different from the episode of PJP, and similar to controls. These results suggested that disruption in the homeostasis of the microbiota by PJP could be restore balance after treatment, but the effect on graft lung function remains unknown. In spite of this, LTRs after PJP recovery had a higher abundance of *Human gammaherpesvirus 4* compared with the control group, which implied the underlying immunocompromised condition. This result further supported the previous suggestion that lifelong PJP prophylaxis for these population is necessary [41].

There are various factors impacting the microbiota after lung transplantation [3]. The overall composition of the lung microbiota was influenced by the neutrophil counts and associated with differential levels of alveolar cytokines in patients with invasive pulmonary aspergillosis [19]. In our study, we found that lung microbiota was related to age, Cr, TP, ALB, neutrophil, and lymphocyte. These findings are supported by pervious reports showing the diversity and community composition of lung microbiota changes were associated with the physical conditions and immune status [42, 43]. Ageing is associated with a dysbiosis and loss of resilience of the resident microbiota in the lungs [44]. As PJP is correlated with immune status but not directly with age, further studies are needed to investigate whether changes in lung microbiota are involved in PJP pathogenesis in elderly patients. Serum TP and ALB levels are important markers of the nutritional and immune state of a patient, that had some potential in predicting the development of PJP [45]. It is also possible that the negative correlation between PJP and ALB is due to the depletion of infection. As nutrition is one of the most accessible modifiable factors affecting microbiota [44], whether hypoproteinemia would promote the occurrence and development of PJP by disturbing the balance of pulmonary microbial flora warrants further study. It is known that the relationship between renal function and gut microbiota dysbiosis is bidirectional, but there is few data about the relationship between renal function and lung microbiota [46]. Previous studies have proved that lung microbiota could predict outcomes in critically ill patients and immunocompromised patients with invasive pulmonary aspergillosis [18, 19]. We could not confirm whether the lung microbiota can predict the prognosis of PJP patients in this study due to the limited sample size. Further research is required to clarify this point.

Our study has certain limitations. First, we cannot conclude whether the alterations in the lung microbiota are a cause or a consequence of the PJP or merely coincide with the disease status. Second, our cohort included LTRs with different underlying diseases, types of lung transplantation, and the PJP group may be affected by coinfection with bacteria and the short-term use of antibiotics before sampling, which cannot be avoided in clinical settings. These clinical heterogeneity factors possibly affecting the results. Third, mNGS was only performed at the DNA level, mainly related to bacteria. Moreover, it cannot distinguish between live and dead microorganisms, and whether or not changes occur in mycobiome and the virome remains to be addressed. Finally, the number of patients was small; therefore, the results must be validated with larger cohort studies.

In conclusion, this was the first study to investigate the lung microbiota in LTRs with or without *P. jirovecii* infection. Our results demonstrated differences in the microbial genera of PJP, compare to PJC and controls. Most species correlated positively with each other, while *P. jirovecii* correlated negatively with other species, suggesting that bacterial dysbiosis and growth after *P. jirovecii* infection. Furthermore, lung microbiota is related to age, renal function, nutritional and immune status. The results of this study would serve as a reference for further studies on the role of microbiota in the transplanted lungs with fungal infection.

### Electronic supplementary material

Below is the link to the electronic supplementary material.

**Fig. S1**. Violin plot shows the differential flora among the PJP, PJC and control groups

**Fig. S2**. Relative abundances of the 15 most abundant microbiota at the genus level in LTRs with PJP and after PJP recovery

**Fig. S3**. LDA identified the most differentially abundant microbiota taxon in PJP, after PJP recovery and control groups. (LDA score > 2.0 with *p*< 0.05).

Supplementary Material 4

## Data Availability

Sequence data that support the findings of this study have been deposited in the NCBI BioProject database (https://www.ncbi.nlm.nih.gov/bioproject) with the primary accession code PRJNA1033798.

## Abbreviations

ALB: Albumin
ALT: Alanine aminotransferase
AST: Aspartate transferase
BALF: Bronchoalveolar lavage fluid
BMI: Body mass index
BUN: Blood urea nitrogen
Cr: Creatinine
DB: Direct bilirubin
FK506: Tacrolimus
GLU: Glucose
HGB: Hemoglobin
LTRs: Lung transplant recipients
LYM: Lymphocyte
mNGS: Metagenomic next-generation sequencing
NEUT: Neutrophil
PJC: *Pneumocystis jirovecii* colonization
PJP: *Pneumocystis jirovecii* pneumonia
PLT: Platelet
TB: Total bilirubin
TMP-SMX: Trimethoprim-sulfamethoxazole
TP: Total protein
WBC: White blood cell
