# Supplementary figures and images for: Alterations of lung microbiota in lung transplant recipients with pneumocystis jirovecii pneumonia
Source: Respir Res. 2024 Mar 14;25:125. doi: 10.1186/s12931-024-02755-9 (PMC10941442; doi:10.1186/s12931-024-02755-9)

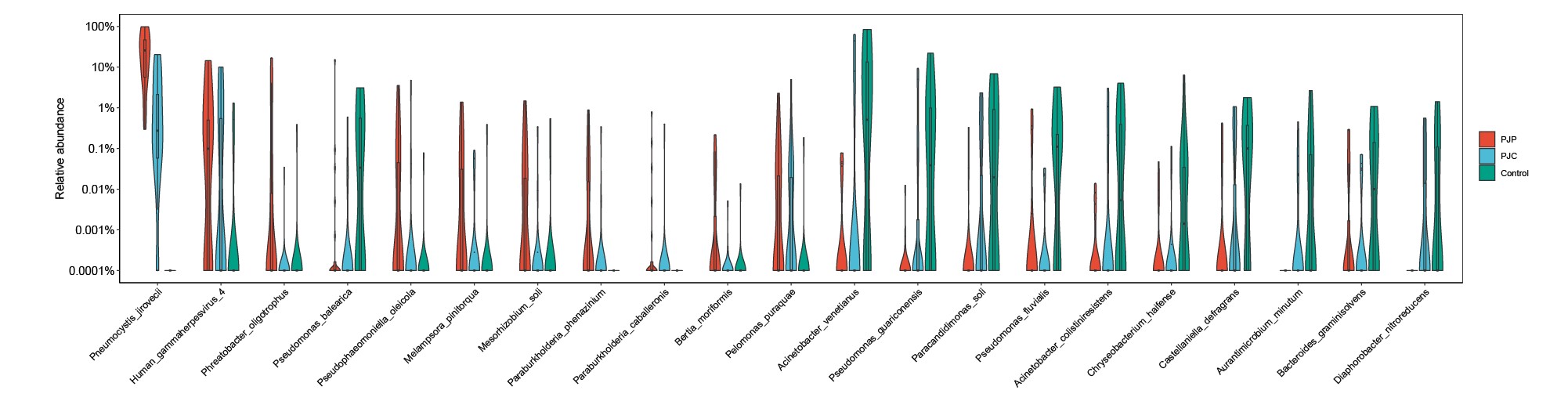

Supplement: Supplementary file 1 — Fig. S1. Violin plot shows the differential flora among the PJP, PJC and control groups [file 12931_2024_2755_MOESM1_ESM.jpeg]

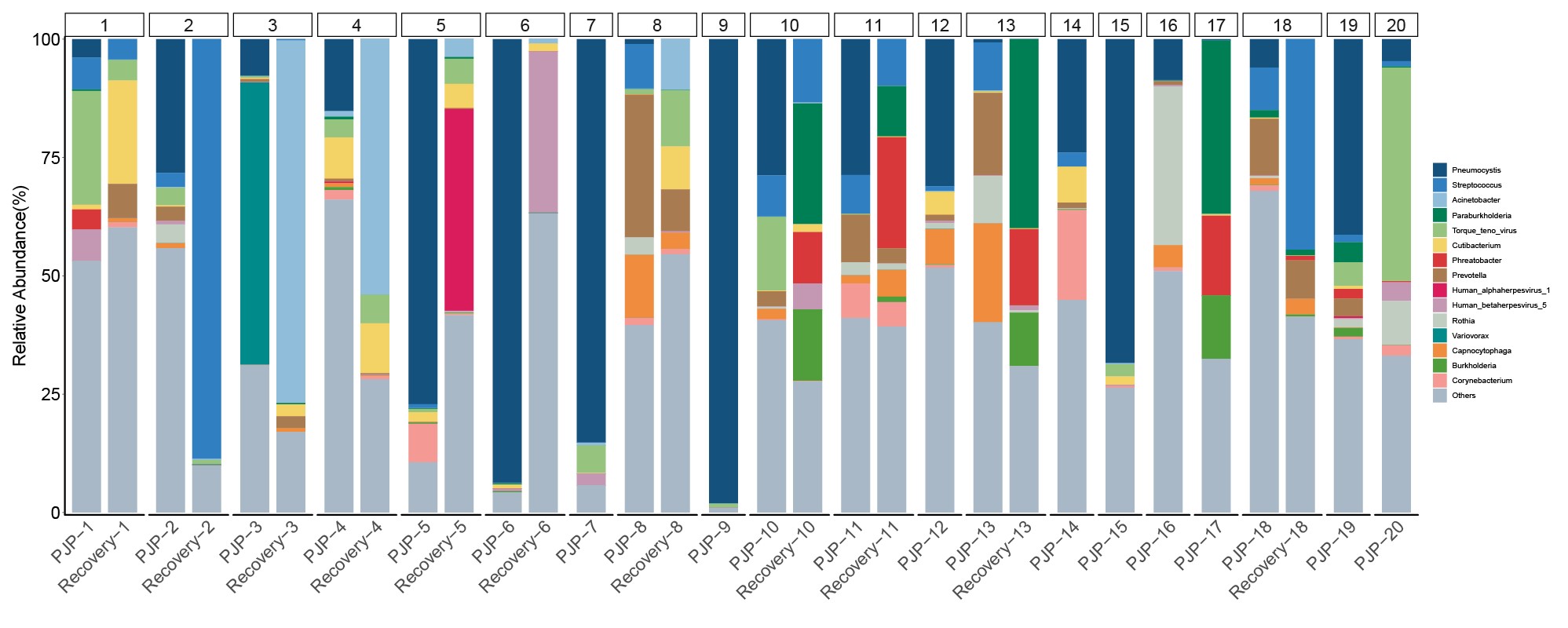

Supplement: Supplementary file 2 — Fig. S2. Relative abundances of the 15 most abundant microbiota at the genus level in LTRs with PJP and after PJP recovery [file 12931_2024_2755_MOESM2_ESM.jpeg]

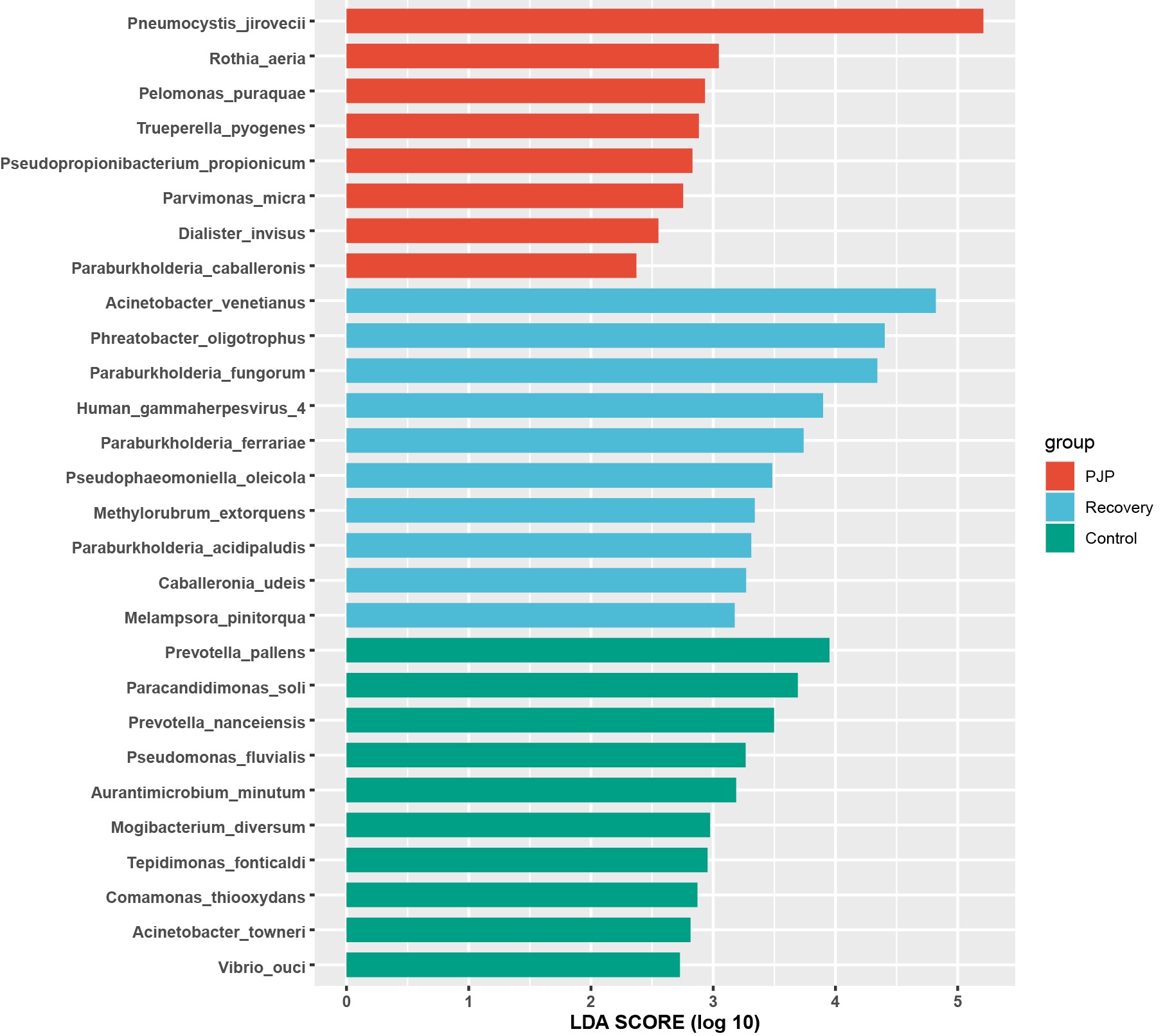

Supplement: Supplementary file 3 — Fig. S3. LDA identified the most differentially abundant microbiota taxon in PJP, after PJP recovery and control groups. (LDA score > 2.0 with p< 0.05). [file 12931_2024_2755_MOESM3_ESM.jpeg]
