# Supplementary material for: Alterations of lung microbiota in lung transplant recipients with pneumocystis jirovecii pneumonia
Source: Respir Res. 2024 Mar 14;25:125. doi: 10.1186/s12931-024-02755-9 (PMC10941442; doi:10.1186/s12931-024-02755-9)
Supplement: Supplementary file 4 — Supplementary Material 4 [file 12931_2024_2755_MOESM4_ESM.docx]

**Table S1** Antibiotic and anti-virus medications of LTRs in PJP group before sampling

| LTRs in PJP group | Indication for lung transplant | Time since lung transplant（day） | Antibiotics | Anti-virus drugs | the medication duration（day） |
| --- | --- | --- | --- | --- | --- |
| Patient 1 | pneumoconiosis | 455 | meropenem | ganciclovir | 1 |
| Patient 2 | pneumoconiosis | 821 | meropenem | ganciclovir | 3 |
| Patient 3 | ILD | 492 | piperacillin-tazobactam | ganciclovir | 1 |
| Patient 4 | ILD | 471 |  |  |  |
| Patient 5 | ILD | 935 | piperacillin-tazobactam |  | 7 |
| Patient 6 | BOS | 355 |  |  |  |
| Patient 7 | ILD | 183 | piperacillin-tazobactam | ganciclovir | 5 |
| Patient 8 | ILD | 222 |  |  |  |
| Patient 9 | COPD | 376 | piperacillin-tazobactam | ganciclovir | 6 |
| Patient 10 | PAP | 246 |  |  |  |
| Patient 11 | ILD | 303 |  |  |  |
| Patient 12 | COPD | 1421 | piperacillin-tazobactam |  | 1 |
| Patient 13 | BOS | 1170 |  |  |  |
| Patient 14 | COPD | 2122 | piperacillin-tazobactam | ganciclovir | 2 |
| Patient 15 | paraquat lung | 495 | piperacillin-tazobactam |  | 2 |
| Patient 16 | pneumoconiosis | 862 | meropenem | ganciclovir | 2 |
| Patient 17 | ILD | 189 | piperacillin-tazobactam | ganciclovir | 4 |
| Patient 18 | ILD | 374 | piperacillin-tazobactam |  | 1 |
| Patient 19 | COPD | 816 |  |  |  |
| Patient 20 | ILD | 363 | piperacillin-tazobactam | ganciclovir | 2 |

BOS, bronchiolitis obliterans syndrome; COPD, chronic obstructive pulmonary disease; ILD, interstitial lung disease; LTRs, lung transplant recipients; PAP, pulmonary alveolar proteinosis; PJP, pneumocystis jirovecii pneumonia.
